# Air as a Stimulant

**Published:** 1879-12

**Authors:** 


### AIR AS A STIMULANT.

The exciting and stimulating properties of pure oxygen are well known, and every one has felt the invigorating influence of fresh air, yet no practical application has been made of these beneficial properties of a substance as cheap and universal. When the body is weak, the brain fatigued, and the whole system in a state of lassitude, just go into the open air, take a few vigorous inspirations and expirations, and the effect will be instantly perceived. The individual trying the experiment will feel invigorated and stimulated, the blood will course with freshness, the lungs will work with increased activity, the whole frame will feel revivified, and nature's stimulant will be found the best.—*Scientific American.*

